# Exploring the relationship between cognitive function and frailty in middle-aged and older adults Chinese adults: evidence from the CHARLS database

**DOI:** 10.3389/fpubh.2026.1825820

**Published:** 2026-05-01

**Authors:** Biqi Zu, Ting Wang, Guangchuan Li, Yajin Chen, Libin An, Juan Yin, Wentao Li

**Affiliations:** 1 Department of Psychiatry, Dalian Seventh People's Hospital, Dalian, China; 2 Department of Research, School of Nursing, Dalian University, Dalian, China

**Keywords:** aging, CHARLS, cognitive impairment, frailty, middle-aged and older adults

## Abstract

**Background:**

Frailty is a complex age-related clinical syndrome characterized by diminished physiological reserve and increased vulnerability to stressors. Cognitive function may be associated with frailty; however, evidence from nationally representative Chinese populations remains limited.

**Methods:**

This cross-sectional study utilized data from the China Health and Retirement Longitudinal Study (CHARLS), a nationally representative survey of individuals aged 45 and older. Cognitive function was assessed using the Mini-Mental State Examination (MMSE), and frailty was evaluated using the Physical Frailty Phenotype (PFP). Data from 19,307 participants were analyzed using logistic regression, Lasso regression, and overlap weighting methods to control for confounding factors. Subgroup analyses by age and gender were conducted to examine variations in the association between cognitive function and frailty.

**Results:**

Among the participants, 13.49% were assessed as having cognitive impairment, and 3.53% were identified as frail. Logistic regression showed that cognitive impairment was associated with a 146% increased risk of frailty (OR: 2.46, 95% CI: 2.15–2.83). Subgroup analysis revealed a stronger association in individuals over 60 years old (OR: 3.35, 95% CI: 2.23–5.06) and in women (OR: 3.25, 95% CI: 1.84–5.74) compared to men (OR: 2.56, 95% CI: 2.03–3.26). Sensitivity analyses confirmed the robustness of these findings.

**Conclusion:**

Cognitive impairment is significantly associated with an increased risk of frailty, particularly in older adults and women. These findings suggest that cognitive decline is associated with higher frailty risk, and this association may be explained by physiological, psychological, and social factors. Future longitudinal and intervention studies are needed to determine whether addressing cognitive impairment can reduce frailty risk and improve health outcomes in aging populations, particularly in vulnerable subgroups.

## 1 Introduction

Frailty, a complex age-related clinical condition characterized by diminished physiological reserve and increased vulnerability to stressors, is a critical geriatric syndrome associated with adverse health outcomes, including disability, hospitalization, and mortality (1, 2). With the rapid population aging in China, frailty has become a pressing public health concern, particularly among adults aged 45 and older. Recent studies estimate that approximately 85.96% of middle-aged and older adults in China' s eastern, central, and western regions are in a pre-frail or frail state, with the prevalence increasing markedly with advancing age (3). Given its profound implications for healthcare systems and quality of life, identifying modifiable risk factors for frailty is essential to developing targeted interventions.

Cognitive function may be closely associated with frailty, yet this relationship remains underexplored (4, 5). Cognitive decline, encompassing impairments in memory, executive function, and processing speed, is common in aging populations and has been linked to physical deterioration (6, 7). Emerging evidence suggests that poorer cognitive performance is associated with slower gait speed, reduced muscle strength, and lower physical activity- key components of frailty (6). Additionally, shared biological mechanisms, such as sarcopenia and neurodegenerative changes, may contribute to the co-occurrence of cognitive impairment and frailty, further supporting their potential interconnection (8, 9).

The relationship between cognitive function and frailty is particularly salient in the Chinese context, where demographic aging and shifting social structures may exacerbate health vulnerabilities (4). Previous studies have primarily focused on Western populations or isolated cognitive domains, leaving gaps in understanding how overall cognitive function influences frailty in Chinese adults (4, 10, 11). While some research has examined frailty in relation to dementia or severe cognitive impairment, fewer studies have investigated milder cognitive deficits, which may be associated with early frailty. Furthermore, existing studies often rely on small or non-representative samples, limiting generalizability (5, 12).

In China, studies in middle-aged and older adults have shown that the prevalence of cognitive impairment ranges from 13.5 to 36.8% (13, 14), the prevalence of frailty is approximately 23.1% (15). Several studies have reported a substantial burden of cognitive impairment and frailty among middle-aged and older adults (16–18). Previous research has suggested that poorer cognitive performance may be associated with frailty or related adverse outcomes. However, many existing studies were based on regional or disease-specific samples (19), focused on limited cognitive domains (20), or did not adequately address confounding and subgroup heterogeneity (21). Evidence from nationally representative Chinese populations remains relatively limited, particularly regarding age- and sex-specific differences in the association between cognitive function and frailty.

Addressing these gaps is crucial for understanding the nature of the association between cognitive function and frailty. The China Health and Retirement Longitudinal Study (CHARLS) offers a unique opportunity to explore this relationship in a nationally representative sample of middle-aged and older adults Chinese adults. Leveraging CHARLS data, this study aims to examine the association between cognitive function and frailty. By clarifying this connection, our findings may provide evidence to support the development of early screening strategies and integrated interventions targeting both cognitive and physical health in aging populations.

## 2 Data and methods

### 2.1 Data source

CHARLS is a nationally representative, longitudinal survey aimed at examining the health, economic, and retirement status of Chinese individuals aged 45 years and older, along with their spouses. Conducted from 2011 to 2020, CHARLS stands as the first national survey focused on the middle-aged and older adults population in China, providing a comprehensive, high-quality public micro-database that is valuable for scientific and policy research.

This study is a cross-sectional analysis based on the 2015 CHARLS data, with ethical approval obtained from the Biomedical Ethics Committee of Peking University (Approval No: IRB00001052-110155). All participants who consented to take part in the survey signed an informed consent form. The CHARLS survey excludes individuals residing in collective dwellings, such as nursing homes, military bases, and schools, ensuring that the data reflect the experiences of community residents.

### 2.2 Inclusion and exclusion criteria

**Figure 1:**
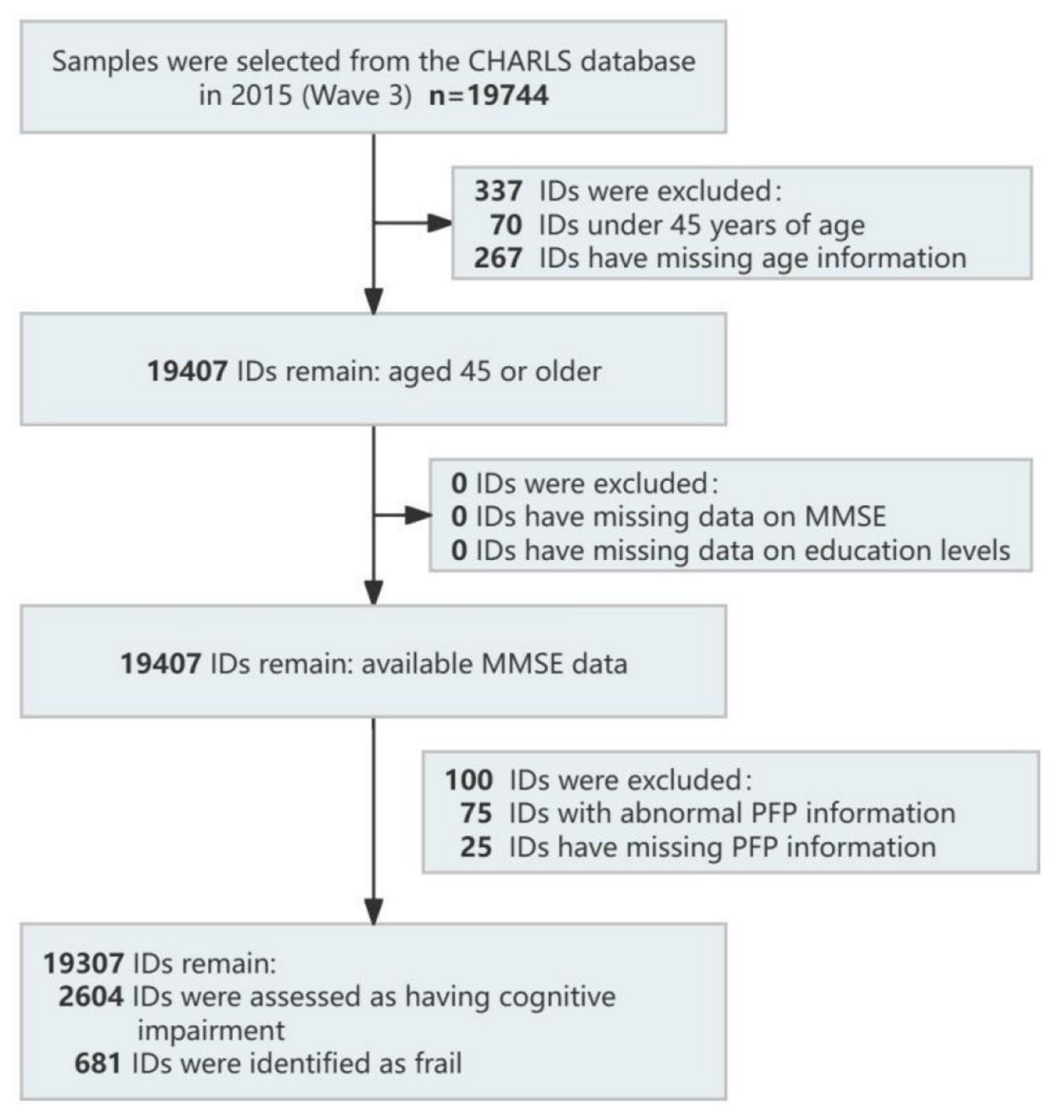
Data filtering flowchart.

Inclusion criteria were age 45 years and above, complete Mini-Mental State Examination (MMSE) data, and complete data required to construct the Physical Frailty Phenotype (PFP). Exclusion criteria were missing age data, missing education level data, and implausible PFP values. The data cleaning process is shown in Figure 1.

### 2.3 Variable definitions

#### 2.3.1 Construction of the PFP

Frailty phenotype. The presence of frailty in middle-aged and older adults was assessed using the Physical Frailty Phenotype (PFP) scale developed by Fried et al. (22), which comprises five criteria: weakness, slowness, exhaustion, low physical activity, and shrinking. Each criterion scores 1 point (total 0–5). Participants were classified as non-frail (0), pre-frail (1–2), or frail (3–5). In the primary analysis, participants classified as pre-frail were grouped with non-frail individuals to create a binary frailty outcome (frail vs. non-frail/pre-frail), consistent with some previous studies focusing on clinically manifest frailty (23). All variables were extracted directly from the CHARLS database. In accordance with prior CHARLS-based frailty studies, the five criteria were operationalized as follows (24).

(1) Weakness: in the CHARLS interview, grip strength was measured by asking participants to squeeze a dynamometer with maximal effort for 3 s. Both hands were measured twice, with >15 s rest between attempts; the maximum value (kg) was used. Weakness was defined as the lowest 20% of maximal grip strength, adjusted for sex and body mass index (BMI).
(2) Slowness: walking speed was assessed by a 2.5-m walk test. Time to complete 2.5 m was used, adjusted for sex and height; the slowest 20% were classified as slowness.
(3) Exhaustion: the items from the CHARLS were used (“I could not get going”). Participants answering either item as “occasionally or a moderate amount of time (3–4 days)” or “most or all of the time (5–7 days)” were defined as exhausted.
(4) Low physical activity: participants who could not walk or perform physical activity for at least 10 min in a typical week were classified as having low physical activity; specific items are in the lifestyle and health behavior section of CHARLS.
(5) Shrinking: weight loss >5 kg in the past year or BMI < 18.5 was defined as shrinking; relevant items are in the health status and functioning section of CHARLS.

#### 2.3.2 Cognitive function

Cognitive function in this study was assessed using the MMSE (25), a widely recognized tool for evaluating cognitive impairment and intellectual functioning. The MMSE provides a comprehensive and accurate reflection of the degree of cognitive deficits and intellectual levels, serving as a basis for neuropsychological diagnosis and treatment. The MMSE covers multiple cognitive domains, including memory, orientation, comprehension, attention, and reading/constructional abilities. The total score ranges from 0 to 30, with higher scores indicating better cognitive function.

In this study, the criteria for cognitive impairment were adjusted based on participants' educational levels: a score of ≤ 17 for illiterate individuals, ≤ 20 for those with primary education, and ≤ 24 for those with secondary education or higher was classified as cognitive impairment, while scores above these thresholds were considered indicative of normal cognitive function.

#### 2.3.3 Depression assessment

Depression: depression symptoms were screened using the 10-item Center for Epidemiological Studies Depression Scale (CESD-10) (26) based on participants' status over the past week (feelings and behaviors from the previous week) in the CHARLS questionnaire. The scale includes ten items (eight negative and two positive). The negative items include: “I worry about little things,” “I have trouble concentrating while doing things,” “I feel sad,” “I feel that everything takes effort,” “I feel afraid,” “My sleep is restless,” “I feel lonely,” and “I feel I cannot go on with my life.” The positive items include: “I have hope for the future” and “I feel good.” Each item has four response options: “rarely or none of the time,” “not very much,” “some of the time or about half the time,” and “most of the time,” which are assigned scores from 0 to 3 (the positive items are reverse scored). The total score ranges from 0 to 30, with higher scores indicating more severe depression symptoms. In this study, depression symptom screening is classified into negative and positive categories, with scores below ten considered harmful and scores of ten or above considered positive for depression symptoms. The CESD-10 has demonstrated good reliability and validity, with a Cronbach's α of 0.78, effectively screening for depression symptoms in the older adults population (27).

## 3 Statistical methods

### 3.1 Statistical description

Continuous measures were summarized as median (IQR) for all samples. Categorical measures were reported as frequency and percentage for crude and matched samples and only for the weighted samples.

### 3.2 Control for confounding factors

Candidate confounders were selected *a priori* based on previous literature, clinical relevance, and data availability in CHARLS, including sex, age, sleep duration, household registration type, education level, marital status, smoking status, drinking status, exercise status, and depression status.

LASSO regression with 10-fold cross-validation was used to identify variables associated with the exposure and/or outcome. Variables with non-zero coefficients at the selected lambda value were retained for propensity score estimation. To minimize the effects of confounding factors, this analysis employed a doubly robust estimation approach, combining overlap weighting and outcome regression, to compare outcomes between the cognitive impairment and non-cognitive impairment groups. Overlap Weighting (OW), a propensity score-based method, was applied to replicate the essential features of randomized controlled trials. This method assigns weights to each sample proportional to the probability of belonging to the opposite treatment group, ensuring the inclusion of all available samples and achieving exact mean balance for all covariates included in the model. Compared to other weighting methods, overlap weighting has demonstrated superior precision in simulation studies.

Multicollinearity among covariates was assessed using variance inflation factors (VIFs).

The newly calculated weights were then integrated into a logistic regression model with Firth's penalization, along with the identified confounders from overlap weighting and additional potential confounding variables. This logistic regression model was used to adjust for confounding effects and assess the impact of cognitive function and frailty. Additionally, stratified analyses by age (<60 vs. ≥60 years) and gender were conducted by fitting the weighted logistic regression model separately within each subgroup (23).

### 3.3 Missing variables and data handling

The dataset exhibited missing values. Multiple imputation methods (28) were used to handle the missing data. Five imputed datasets were constructed based on the data in Table 1, and the Rubin formula (29) was applied to obtain model estimates and to compare the corrected standard errors of the imputations.

**Table 1:** Characteristics of confounding variables and overlap weighting results.

Items	Unweighted			Weighted^b^		
	Cognitive impairment	Non-cognitive impairment	Standardized Mean Differenced^a^	Cognitive impairment	Non-cognitive impairment	Standardized Mean Differenced
Samples, No. (%)	2,604 (13.49)	16,703 (86.51)				
Gender, No. (%) or %						
Male	1,139 (43.74)	7,724 (46.24)	0.201	46.92	47.68	0.015
Female	1,465 (56.26)	8,979 (53.76)		53.08	52.32	
Age, median (IQR)	62.00 (54.00–69.00)	60.00 (53.00–67.00)	0.819	60.00 (54.00–67.00)	61.00 (53.00–67.00)	0.469
Sleep duration, median (IQR)	6 (5–7)	6 (5–8)	0.048	6 (5–7)	6 (5–7)	0.036
Household registration type, no. (%) or %						
Rural	1,797 (69.01)	12,512 (74.91)	0.144	72.78	73.99	0.045
Urban	602 (23.12)	2,919 (17.48)		20.09	18.39	
Others	205 (7.87)	1,272 (7.62)		7.14	7.62	
Education level, no. (%) or %						
Primary school or below	1,343 (51.57)	6,920 (41.43)	0.524	47.91	42.99	0.102
Junior high school	851 (32.68)	3,470 (20.77)		21.31	22.35	
High school or above	410 (15.75)	6,313 (37.80)		30.79	34.66	
Marital status, no. (%) or %						
Married	2,232 (85.71)	14,317 (85.72)	0.019	79.49	85.56	0.162
Unmarried	12 (0.46)	99 (0.59)		0.64	0.58	
Divorced/Widowed	360 (13.82)	2,287 (13.69)		19.87	13.86	
Drinking status, no. (%) or %						
Yes	786 (30.18)	4,291 (25.69)	0.100	23.26	26.15	0.067
No	1,818 (69.82)	12,412 (74.31)		76.74	73.85	
Exercise status, no. (%) or %						
Yes	2,414 (92.70)	15,003 (89.82)	0.102	87.26	90.17	0.092
No	190 (7.30)	1,700 (10.18)		12.74	9.83	
Depression status, no. (%) or %						
Yes	841 (32.30)	6,734 (40.32)	0.167	42.14	39.28	0.036
No	1,763 (68.70)	9,969 (59.68)		57.86	60.72	

a Absolute value of the between-group difference in means or proportions divided by the pooled SMD.

b After overlap weighting, a single sample No longer represents a single data entity and thus raw counts are Not reported after overlap weighting.

### 3.4 Sensitivity analysis

Propensity Score Matching (PSM) was used for sensitivity analysis, employing nearest neighbor matching with a caliper value of 0.2 to further validate the effect size obtained from the logistic regression model. Additionally, to evaluate the robustness of the observed association between cognitive function and frailty, the E-value was calculated following the method proposed by VanderWeele and Ding (30).

A two-sided significance level of 0.05 was considered statistically significant. Odds ratios (OR) with 95% confidence intervals (CIs) were reported. Due to the exploratory nature of the study and the potential for type I errors caused by multiple comparisons, all findings should be interpreted cautiously. All statistical analyses were performed using R software version 4.3.3.

## 4 Results

### 4.1 General results

A total of 19,307 participants were included in the study, of which 8,863 were male, accounting for 45.90% of the total sample. Among the participants, 2,604 were assessed as having cognitive impairment, representing approximately 13.49% (2,604/19,307) of the total sample. A total of 681 participants were identified as frail, accounting for 3.53% (681/19,307) of the total sample, 64.51% (12,456/19,307) samples were identified as pre-frail. The overall data had a low proportion of missing values, as follows: sleep duration had five missing values (0.02%), household registration type had 28 missing values (0.15%), smoking status had eight missing values (0.04%), and depression status had 2,288 missing values (11.85%).

### 4.2 Lasso regression and overlap weighting results

**Figure 2:**
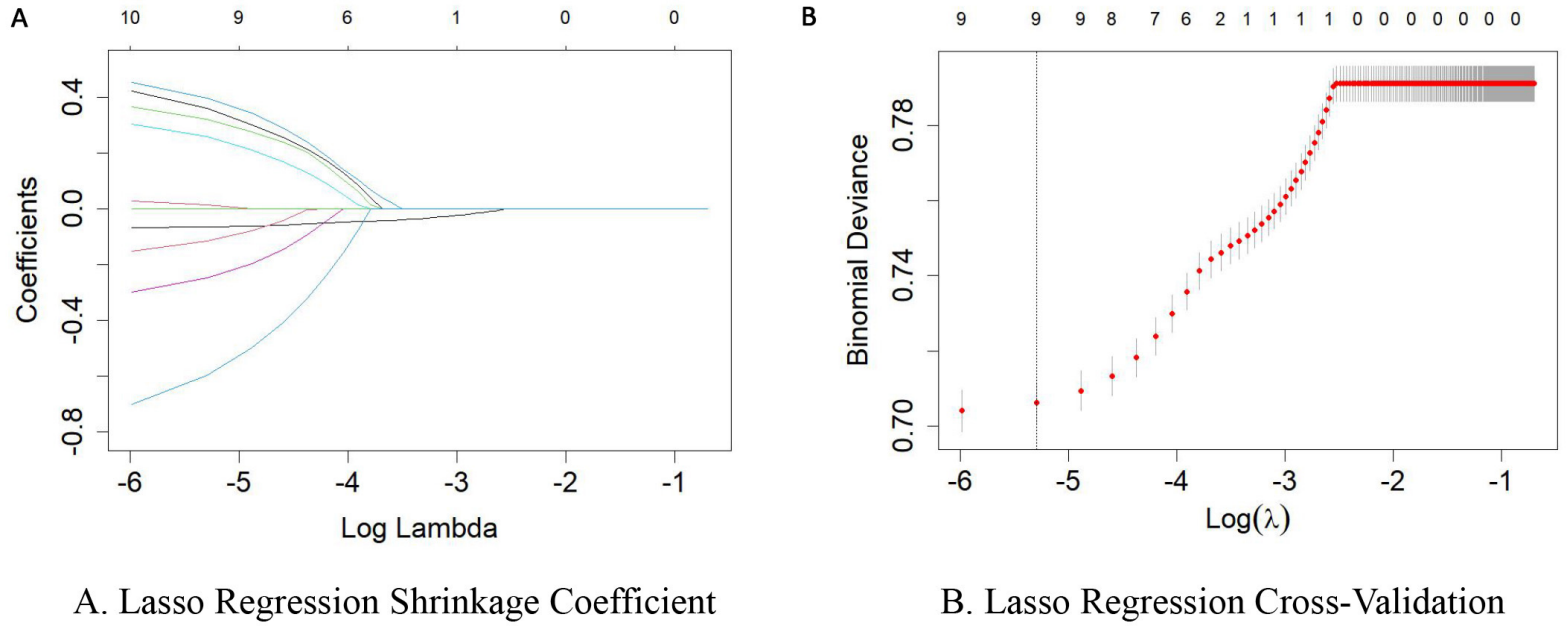
Lasso regression potential confounder selection plot. **(A)** Lasso Regression Shrinkage Coefficient. **(B)** Lasso Regression Cross-Validation.

Based on previous literature and expert knowledge, 10 variables- gender, age, sleep duration, household registration type, education level, marital status, smoking status, drinking status, exercise status, and depression- were included as potential confounders in the Lasso regression analysis to identify confounders highly related to the outcome. The binomial deviance was minimized when λ= 0.00502, at which point the coefficient for smoking status was reduced to zero, while the remaining nine variables were highly correlated with the outcome (Figure 2). To reduce inter-group differences, overlap weighting was applied to these nine variables. The results showed that, except for marital status, the standardized mean differences for the other variables were reduced after applying overlap weighting (Table 1).

### 4.3 Logistic regression and sensitivity analysis results for the association between cognitive function and frailty

Collinearity diagnostics showed that the VIFs values for all variables ranged from 1.052 to 1.486, indicating no substantial multicollinearity. Weights were incorporated into a binary logistic regression model to explore the relationship between cognitive function and frailty (Table 2). The results showed an odds ratio (OR) of 2.46 (95% confidence interval [CI]: 2.15–2.83), indicating that individuals with cognitive impairment had a 146% higher risk of frailty compared to those with normal cognitive function. Propensity score matching (PSM) and E-value were calculated to evaluate the robustness of the observed association between cognitive function and frailty. The PSM results showed an OR of 1.74 (95% CI: 1.16–2.66; Table 3), and the E-value was 3.12, with a lower bound of the confidence interval of 2.78. This suggests that the results remain robust unless unmeasured confounders have an OR greater than 2.78 that could invalidate the findings, indicating the stability of the results.

**Table 2:** Multivariate logistic regression analysis after overlap weighting.

Variables	*B*	SE	Wald χ^2^	OR (95% CI)	*P* value
Cognitive impairment	0.90	0.06	175.35	2.46 (2.15, 2.83)	<0.01
Age	0.06	0.01	197.31	1.06 (1.05, 1.07)	<0.01
Sleep duration	0.01	0.02	1.09	1.01 (0.98, 1.05)	0.29
Education level					
*Primary school and below*	–	–	–	–	
*Middle school*	−0.24	0.12	3.99	0.78 (0.61, 0.99)	0.04
*High school or above*	−0.45	0.13	11.63	0.63 (0.48, 0.82)	<0.01
Marital status					
*Married*	–	–	–	–	
*Unmarried*	0.27	0.43	0.39	1.31 (0.56, 3.08)	0.53
*Divorced/Widowed*	−0.15	0.11	1.96	0.85 (0.68, 1.06)	0.16
Household registration type					
*Rural*	–	–	–	–	
*Urban*	0.16	0.13	1.55	1.18 (0.90, 1.54)	0.21
*Other*	0.41	0.17	5.38	1.51 (1.06, 2.13)	0.02
Depression status (YES)	1.71	0.11	245.94	5.57 (4.49, 6.90)	<0.01
Drinking status (YES)	−1.00	0.16	37.32	0.36 (0.26, 0.50)	<0.01
Exercise status (YES)	−2.18	0.09	581.54	0.11 (0.09, 0.13)	<0.01

OR, odds ratio; “-”, reference.

**Table 3:** Logistic regression analysis of the association between cognitive function and frailty under different models.

Method	*N*	*B*	SE	Wald χ^2^	OR (95% CI)	*P* value
Model 1 (original data)	19,307	1.05	0.17	37.20	2.87 (2.04, 4.02)	<0.01
Model 2 (PSM)	5,208	0.55	0.21	6.89	1.74 (1.16, 2.66)	0.09
Model 3 (OW)	^*^	0.90	0.07	164.96	2.46 (2.15, 2.83)	<0.01

^*^, After overlap weighting, the weighted sample sizes are not integers and therefore cannot be presented.

### 4.4 Subgroup analysis results

#### 4.4.1 Age subgroup analysis

In the subgroup of participants aged under 60 years, the relationship between cognitive function and frailty showed an OR of 0.40 (95% CI: 0.05–5.15), which was not statistically significant. In contrast, in the subgroup of participants aged over 60 years, the relationship between cognitive function and frailty showed an OR of 3.35 (95% CI: 2.23–5.06), indicating that cognitive impairment in older individuals was associated with a higher risk of frailty (Table 4).

**Table 4:** Logistic regression analysis of the association between cognitive function and frailty across age and sex subgroups under different models.

Model	*B*	SE	Wald χ^2^	OR (95% CI)	*P* value
Age subgroup					
*<60*					
Model 1 (original data)	−1.38	0.85	2.61	0.25 (0.13, 3.83)	0.98
Model 2 (PSM)	−0.96	0.91	1.11	0.38 (0.12, 4.35)	0.98
Model 3 (OW)	−0.90	1.17	0.60	0.40 (0.05, 5.15)	0.95
*≥60*					
Model 1 (original data)	1.56	0.17	81.59	4.76 (3.39, 6.69)	<0.01
Model 2 (PSM)	1.25	0.18	47.57	3.52 (2.45, 5.04)	<0.01
Model 3 (OW)	1.21	0.20	33.93	3.35 (2.23, 5.06)	<0.01
Gender subgroup					
*Male*					
Model 1 (original data)	0.90	0.22	15.80	2.47 (1.58, 3.86)	<0.01
Model 2 (PSM)	1.04	0.24	18.17	2.85 (1.75, 4.62)	<0.01
Model 3 (OW)	094	0.12	60.58	2.56 (2.03, 3.26)	<0.01
*Female*					
Model 1 (original data)	1.18	0.26	20.10	3.28 (1.95, 5.52)	<0.01
Model 2 (PSM)	1.05	0.27	14.51	2.85 (1.66, 4.90)	<0.01
Model 3 (OW)	1.18	0.29	16.51	3.25 (1.84, 5.74)	<0.01

#### 4.4.2 Gender subgroup analysis

In the male subgroup, the association between cognitive function and frailty showed an OR of 2.56 (95% CI: 2.03–3.26). However, in the female subgroup, the OR was 3.25 (95% CI: 1.84–5.74), indicating that women with cognitive impairment had a higher risk of frailty (Table 4).

## 5 Discussion

This study, based on a large nationally representative sample of middle-aged and older Chinese adults, provides robust evidence that cognitive impairment is significantly associated with frailty. Compared with previous studies, our work extends the existing literature by using a nationally representative Chinese dataset, applying overlap weighting and sensitivity analyses, and further examining heterogeneity by age and gender. These methodological features enhance both the generalizability and the robustness of the findings.

Importantly, the observed association remained stable after adjustment for a broad range of demographic, socioeconomic, behavioral, and health-related factors, suggesting that it is unlikely to be fully explained by measured differences in baseline characteristics such as age, gender, education, marital status, smoking, drinking, or chronic disease burden. Given that cognitive impairment and frailty are both multifactorial geriatric syndromes with overlapping determinants, this finding supports the presence of a relatively independent link between the two conditions.

The weighted analyses further strengthened this interpretation. By improving covariate balance between participants with and without cognitive impairment, overlap weighting allowed for a more comparable assessment of the association and reduced the influence of measured confounding. The consistency of the results across conventional regression, weighting, and sensitivity analyses indicates that the association is not merely an artifact of model specification or group imbalance, but rather a relatively robust finding. Nevertheless, although these methods improve comparability with respect to measured covariates, residual confounding from unmeasured or imperfectly measured factors cannot be completely ruled out.

### 5.1 The significant relationship between cognitive impairment and frailty

The present study adds to the growing body of evidence indicating that cognitive impairment and frailty are closely interconnected in middle-aged and older adults. Consistent with previous studies, including that of Dong et al. (16), our findings support the view that poorer cognitive function is associated with a greater likelihood of frailty. However, rather than merely confirming an existing association, this study provides several important extensions to the current literature.

First, compared with many previous studies based on regional samples or specific clinical populations, this analysis was conducted using data from the CHARLS, a nationally representative cohort of middle-aged and older adults in China. This improves the generalizability of the findings and provides population-level evidence for the association between cognitive impairment and frailty in the Chinese context. Second, we applied multiple analytical strategies to improve the robustness of the findings, including conventional regression analysis as well as propensity score-based and weighting approaches to better control for potential confounding. The persistence of the association across these different models strengthens confidence that the observed relationship is not solely attributable to imbalance in measured baseline characteristics. Third, by further examining age and sex subgroups, this study highlights potential heterogeneity in the cognitive function–frailty association, an aspect that has not been sufficiently explored in many previous studies.

Several mechanisms may explain the close relationship between cognitive impairment and frailty. From a biological perspective, both conditions may share common pathological pathways, including neurodegeneration, chronic inflammation, oxidative stress, endocrine dysregulation, and vascular damage (31–34). These processes may simultaneously impair brain function and reduce physiological reserve, thereby increasing vulnerability to frailty. In addition, cognitive impairment is often accompanied by deficits in memory, executive function, and attention, which may reduce an individual's ability to maintain medication adherence, adequate nutrition, regular exercise, and other health-promoting behaviors. Over time, these changes may accelerate functional decline and contribute to the development of frailty.

Psychological and social pathways may also play an important role. Individuals with cognitive impairment are more likely to experience depressive symptoms, reduced motivation, and social withdrawal (35, 36). These factors may lead to lower levels of physical activity, less social participation, and diminished engagement in daily activities, all of which are closely related to muscle loss, reduced mobility, and worsening frailty status. Furthermore, cognitive impairment frequently coexists with multimorbidity and sarcopenia, which may further exacerbate physiological vulnerability and adverse health outcomes (37).

Taken together, our findings suggest that the relationship between cognitive impairment and frailty is likely multidimensional, involving overlapping biological, behavioral, psychological, and social mechanisms. The novelty of this study lies not only in confirming this association in a large nationally representative sample, but also in demonstrating its robustness across multiple analytic approaches and exploring subgroup heterogeneity by age and sex. These findings underscore the importance of integrating cognitive assessment into frailty screening and support the development of multidomain interventions targeting both cognitive and physical vulnerability in middle-aged and older adults.

### 5.2 The relationship between cognitive function and frailty differs across age groups

Through subgroup analysis by age, this study found that the relationship between cognitive function and frailty was not significant in individuals under 60 years of age, whereas a stronger association was observed in those over 60 years. As age increases, the relationship between cognitive decline and frailty becomes more pronounced, which is consistent with the findings of Daddimani et al. (38).

The age-related differences in the association between cognitive function and frailty may be explained by physiological and biochemical changes during aging. As individuals age, the degeneration of the nervous system and changes in immune metabolism are associated with cognitive function (39, 40), especially in older populations, where these associations are more pronounced. These physiological changes may explain why frailty is more closely associated with cognitive impairment in older adults. Moreover, chronic diseases in older individuals, such as cardiovascular disease, diabetes, and thyroid disorders, are associated with both cognitive decline and frailty, and may share common underlying mechanisms (41, 42). For individuals under 60 years, although cognitive decline may still occur, the combined influence of physiological, psychological, and social support factors results in a less pronounced relationship between cognitive impairment and frailty compared to older populations (43, 44).

### 5.3 The impact of cognitive function on frailty is more significant in women

In the gender subgroup analysis, the relationship between cognitive impairment and frailty was more significant in women, whereas in men, this association was relatively mild. This is consistent with previous studies (45, 46), suggesting that women may be at higher risk of frailty due to physiological differences (such as hormonal levels and bone density) and higher rates of depression.

Physiologically, the significant decline in hormonal levels after menopause is associated with bone and muscle deterioration in women (47, 48), which may be related to higher vulnerability to frailty in the presence of cognitive impairment. On the other hand, women may also face more pressure and responsibility in their social roles, which could limit their ability to receive adequate support when cognitive function declines. For example, women often play a significant caregiving role in families, but when faced with cognitive decline, they may be unable to effectively fulfill these responsibilities, which may be associated with reduced social participation and more severe frailty symptoms (49). Additionally, the higher prevalence of depression in women may be associated with more severe frailty symptoms (50, 51). Depression is associated with more severe cognitive impairment and lower motivation for physical activity and social engagement, which may be related to higher frailty risk.

### 5.4 Limitations

Despite providing valuable insights into the relationship between cognitive function and frailty in middle-aged and older adults Chinese adults, this study has several limitations that warrant further exploration in future research. This study employs a cross-sectional design, which, due to the lack of time-series data, cannot infer causal relationships. Due to the cross-sectional design, this study can only demonstrate associations between cognitive function and frailty, and cannot establish causality or determine the directionality of the relationship. Longitudinal studies are needed to clarify temporal sequences and causal pathways. Although the study attempts to control for confounding variables using Lasso regression and overlap weighting methods, there may still be potential unmeasured confounders that could affect the results. For example, genetic factors, environmental influences, and other variables not included in the analysis may impact the relationship between cognitive function and frailty. The data used in this study from the CHARLS database is highly representative, but the analysis is limited to community residents, excluding individuals living in institutional settings such as nursing homes, military bases, or schools. This may restrict the generalizability of the findings. Although multiple imputation methods were used to handle missing data, data missingness itself could still introduce bias.

## 6 Conclusion

This study found a significant association between cognitive impairment and frailty, with this association being more pronounced in older adults and women. The findings suggest that cognitive impairment is significantly associated with frailty, and that the nature of this association may differ across subgroups. In older and female populations, the decline in cognitive function is associated with higher frailty risk, and this association may involve physiological, psychological, and social factors. Therefore, future longitudinal and intervention studies should focus on these subgroups to determine whether early interventions for cognitive impairment can reduce frailty incidence.

## Data Availability

The raw data supporting the conclusions of this article will be made available by the authors, without undue reservation.
